# Follow-up cancer care in Danish general practice: a questionnaire study

**DOI:** 10.3399/BJGPO.2023.0215

**Published:** 2024-06-26

**Authors:** Lisa Maria Sele Sætre, Steffi Blach Naamansen, Kirubakaran Balasubramaniam, Jens Søndergaard, Dorte Ejg Jarbøl

**Affiliations:** 1 Research Unit of General Practice, Department of Public Health, University of Southern Denmark, Odense, Denmark

**Keywords:** patient perspectives, cancer, neoplasms, continuity of patient care, general practice

## Abstract

**Background:**

The number of patients who have survived cancer has increased substantially owing to improved cancer treatment. This has reinforced the need for effective strategies for follow-up cancer care in general practice.

**Aim:**

To investigate the organisation of follow-up cancer care in Danish general practice and to analyse GPs’ self-assessment of competences regarding patients who have survived cancer and late effects.

**Design & setting:**

A total of 500 Danish general practices were invited to a web-based survey.

**Method:**

Questions comprised organisation of follow-up cancer care, and GPs’ self-assessment of their competences in follow-up care and evaluation of late effects. Covariates considered included sex, age, seniority, and practice type. Analyses were conducted using descriptive statistics and multivariable logistic regression models.

**Results:**

Some 28% of the GPs reported systematic organisation of follow-up cancer care in their clinic. More than half of the GPs assessed themselves as competent in evaluating mental sequelae, existential considerations, and the impact on comorbidities. In contrast, only 19% and 33% of GPs reported competences in sexual and physical sequelae, respectively. Female GPs were less likely to report competences regarding physical and mental sequelae as well as sexual disturbances, and GPs from partnership practices were more likely to report competence in assessing mental sequelae.

**Conclusion:**

Fewer than one in three general practices have organised systematic follow-up cancer care and GPs assess their competence as low with respect to physical sequelae and sexual challenges. This emphasises the need for more systematic organisation and focus on knowledge of late effects in general practice.

## How this fits in

The increasing number of patients who have survived cancer underscores the demand for follow-up cancer care in general practice. Less than one-third of general practices have organised follow-up care and GPs report insufficient competences in addressing physical sequelae and sexual challenges.

## Introduction

Cancer remains a major cause of disease worldwide, with almost 20 million new cases in 2020.^1^ The combination of an ageing population and improvements in cancer treatment and survival has led to a growing population of patients who have survived cancer worldwide, including Denmark.^2^ Many patients who have survived cancer face late effects for years after the end of cancer treatment, and often even lifelong.^3^ Hence the need for follow-up care is increasing significantly, prompting researchers, healthcare organisers, and patient organisations to suggest expanding and reorganising follow-up cancer care.^4^ The main part of cancer care follow-up has traditionally been dealt with by the hospitals in Denmark, although recently updates from the Danish Health Authority for the cancer follow-up programmes have involved task delegation to general practice for the follow-up of prostate, bladder, and kidney cancer.

Owing to the expanding needs of, for example, accessibility and individualisation, alongside organisational changes in the healthcare sector as a whole, the importance of general practice’s role has been highlighted both nationally and internationally.^2^

A recent review concluded that quality of care for patients with cancer increases when GPs have a proactive approach to the patients; act as interpreters of the cancer diagnosis; assess treatment options and its consequences; and engage in care coordination with specialists in secondary care while keeping the patient’s resources and wishes in mind.^5^ Likewise, the Danish Health Authority highlights the GP’s important role as coordinator throughout the entire course of diagnostics, treatment, and long-term care.^6,7^

The guidelines for follow-up cancer care from the Danish College of General Practitioners recommends implementing systematic efforts in general practice to ensure that all patients with cancer are offered the necessary follow-up, for example, by a systematic proactive approach, regular controls, and assessment of the potential late effects. Most patients with cancer have at least one comorbidity in addition to their cancer disease, which enhances the risk of physical and psychological distress.^8^ This emphasises the need for patient-centred and persistent follow-up care after cancer.

Knowledge on how follow-up cancer care is organised in general practice in Denmark and whether the GPs feel competent to assess, for example, patients’ late effects, is, however, sparse. To ensure the possibilities of a well-structured and well-organised follow-up cancer care in general practice, knowledge about the current organisation and the GP’s qualifications is needed. Thus, the aim of this questionnaire study is to investigate the organisation of follow-up cancer care in Danish general practice and analyse GPs’ self-assessment of competence in addressing late effects, along with the impact on comorbidities and lifestyle.

## Method

### Setting and design

This study is based on a survey conducted in Danish general practices from September–November 2021.

GPs play a pivotal role as gatekeepers and care coordinators, collaborating with both primary and secondary healthcare providers in Denmark. Danish GPs are remunerated through taxes in a mixed capitation and fee-for-service system, and nearly all medical services are provided to the patients free of charge. Regarding patients with cancer, the hospitals possess specialised knowledge about the patient’s cancer treatments, late effects, and prognosis, and the department provides a discharge summary. The GPs follow the patient throughout their life and manage both acute and chronic conditions. Moreover, they provide continuity of care for the patients and have the opportunity to address the psychological, social, and existential consequences of cancer, including concerns about recurrence.

### Participants and sampling

The study population comprised 500 general practices, 250 from the Capital Region of Denmark and 250 from the Region of Southern Denmark, randomly selected by the unique company registration number (CVR number) and invited to participate in a web-based survey about follow-up and end-of-life care of patients with cancer in general practice. The Capital Region of Denmark was selected owing to its status as the largest region in Denmark, with the highest number of citizens and GPs. The Region of Southern Denmark was chosen as it mirrors the general population’s composition in Denmark.

The invitation to the survey was sent to a digital mailbox linked to the CVR number in September 2021. A reminder letter was sent to the digital mailbox encouraging the GPs to participate after 2 weeks and 4 weeks, respectively. Participation was voluntary and an economic compensation corresponding to 20 minutes (approximately 37 €) was offered to each participating GP.

### Questionnaire development

The conceptual framework and constructs in the questionnaire were developed based on existing literature and guidelines regarding follow-up care for patients with cancer in general practice.^5,6^ The definition of follow-up cancer care aligns with the definition in the guideline for follow-up cancer care in general practice from the Danish College of General Practitioners, whereby cancer follow-up encompasses processes where the active treatment of patients with cancer has concluded or where the patient has transitioned to medical aftercare. We framed it as follows in the questionnaire: ‘*Follow-up care for patients who have undergone treatment for cancer includes both follow-up care after the active treatment has ended, and the patient is cancer-free, and follow-up care for patients who receive long-term aftercare for their cancer.*’^6^ The definition therefore includes both patients who have completed their treatment and patients who receive adjuvant, palliative, or no treatment.

Based on the conceptual framework the following four domains were included in the questionnaire: (i) the organisation of follow-up cancer care in general practice; (ii) professional efforts and competences in general practice; (iii) interdisciplinary and cross-sectorial cooperation; and (iv) coordination and coherent processes. For each domain, several items were generated. The questionnaire was assessed and discussed in a multidisciplinary group consisting of a nurse, younger doctors, GPs, and representatives from a patient organisation.

Before the distribution of the questionnaire, two pilot tests were conducted to ensure the content validity. In the first pilot test, the questionnaire was sent to GPs associated with the research environment. This test resulted in reduction of questions and clarifications of the definitions of follow-up cancer care. In the second pilot test, the questionnaire was sent to selected general practices in the two regions, resulting in rewordings and comprehension changes. The final questionnaire included 18 questions: five questions regarding GP characteristics and 13 questions covering the four domains.

In this article, we included questions from the first two domains, which relate to the GP’s management and self-assessment of competences in follow-up cancer care. Management included systematic efforts and contacts. GPs answering that they had implemented systematic efforts for follow-up cancer care had the opportunity to describe their effort in free-text boxes. Self-assessment of competences in follow-up cancer care were related to comorbidities (other somatic diseases and treatments), physical sequelae, sexual sequelae (challenges and problems related to sexuality and intimacy), mental sequelae, social sequelae, existential considerations, and lifestyle challenges. The GP’s perception of follow-up cancer care and self-assessment of competences in late effects was reported on a 5-point Likert scale.

### Covariates and statistical analyses

Owing to the risk of ambiguous information on practice characteristics and organisation of follow-up cancer care, GPs with more than one provider number were excluded before invitation. GPs employed in a regional-owned practice and GPs with incomplete answers to the questionnaire were excluded from analyses.

The GPs’ ratings for management of follow-up cancer care were categorised as follows: very rarely or rarely; sometimes; and often or very often. GPs’ self-assessment of qualifications were categorised as follows: not at all, to a very low degree, or to a low degree; somewhat; and to a high or very high degree. The proportion of answers within each category was calculated by using descriptive statistics, and χ^2^ test were used to test for differences between groups.

The associations between GP- and practice-characteristics and having implemented systematic efforts (yes or no) were analysed by multivariable logistic regression models. GPs’ self-assessment of competences were analysed in multivariable ordered logistic regression models including all outcome variables (not at all, to a very low degree, to a low degree, somewhat, to a high degree, and to a very high degree).

The covariates included in the analyses were categorised as follows: sex (male or female); seniority in practice (<10 years, 10–19 years, ≥20 years); practice type (partnership practice or single-handed practice); and region (Capital Region of Denmark and Region of Southern Denmark). The models were adjusted for sex, seniority, practice type, and region.

Data analyses were conducted using Stata statistical software (version 17). All tests used a significance level of *P* value <0.05.

## Results

**Figure 1.**
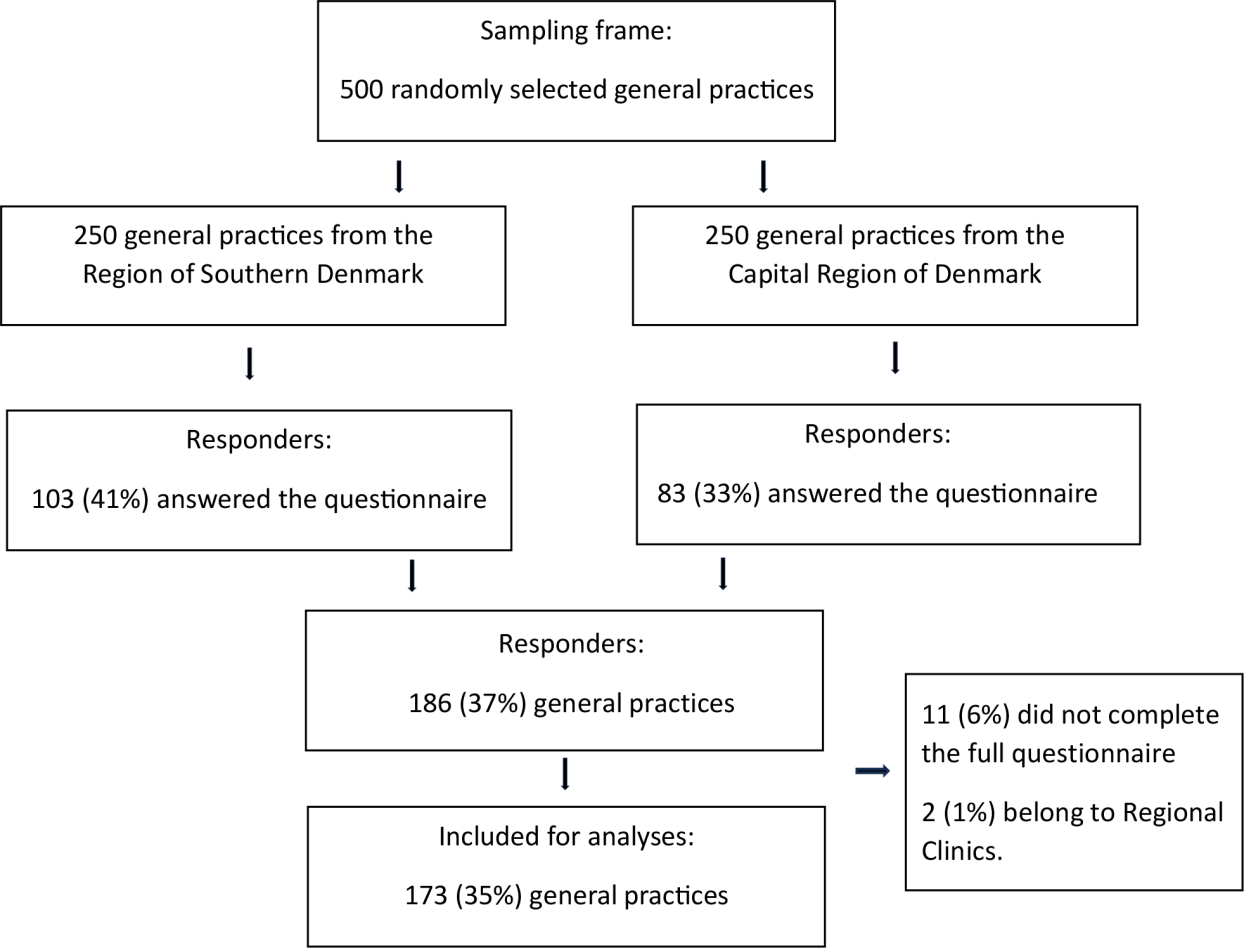
Flow of participants

From the 500 invited general practices, 173 GPs completed the entire questionnaire (35%), as shown in Figure 1.

The distribution of GP and practice characteristics appear in Table 1.

**Table 1.** GP and practice characteristics

Characteristics	Total^a^ *n* (%)	Capital Region of Denmark, *n* (%)	Region of Southern Denmark, *n* (%)	*P* value^b^
Total	173 (100)	76 (43.9)	97 (56.1)	
Sex				0.25
Female	94 (54.3)	45 (59.2)	49 (50.5)	
Male	79 (45.7)	31 (40.8)	48 (49.5)	
Mean age, years (range)	53 (32–72)	52 (32–72)	54 (37–70)	
Age groups				
30–39 years	11 (6.4)	3 (3.9)	8 (8.2)	0.62
40–49 years	57 (32.9)	24 (31.6)	33 (34.0)	
50–59 years	60 (34.7)	27 (35.5)	33 (34.0)	
≥60 years	45 (26.0)	22 (28.9)	23 (23.7)	
Seniority in practice				
<10 years	56 (32.4)	24 (31.6)	32 (33.0)	0.95
10–19 years	60 (34.7)	26 (34.2)	34 (35.1)	
≥20 years	57 (32.9)	26 (34.2)	31 (32.0)	
Practice type				
Partnership practice	96 (55.5)	36 (47.4)	60 (61.9)	0.06
Single-handed practice	77 (44.5)	40 (52.6)	37 (38.1)	

^a^National data 2021: women = 58%; men = 42%. Mean age = 51.5 years. Practice type: partnership = 59%; single-handed = 41%. ^b^χ^2^ test for difference between the two regions.

The GPs most often received information about patients’ need for follow-up through discharge letters from the hospital (49%) or from the patients or relatives (47%). Some 41% of the GPs contacted the patients in writing or by telephone encouraging them to contact the practice, if necessary (Table 2).

**Table 2.** GPs’ perceptions of follow-up cancer care (*n* = 173)

	Very rarely or rarely *n* (%)	Sometimes *n* (%)	Frequently or very frequently *n* (%)
How often do you identify patients in need of follow-up cancer care in your practice by:			
Reaching out to the patients with cancer through written communication or telephone, and encouraging them to contact the practice if necessary?	40 (23.1)	62 (35.8)	71 (41.0)
Contacting patients through written communication or by telephone to offer consultation in practice?	45 (26.0)	65 (37.6)	63 (36.4)
Receiving information through discharge cards or by telephone from the hospital regarding patients’ requiring follow-up?	35 (20.2)	53 (30.6)	85 (49.1)
Receiving information through written communication or telephone from the patients’ home municipality?	80 (46.2)	49 (28.3)	44 (25.4)
Being contacted in writing or by telephone by a patient or relative?	18 (10.4)	74 (42.8)	81 (46.8)
	**Not at all or very low or low** ***n*** **(%)**	**Somewhat** ***n*** **(%)**	**Highly or very highly** ***n*** **(%)**
To what extent do you feel qualified to assess the following possible sequelae after the patients’ cancer diagnosis and treatment:			
Other somatic disease and treatment?	5 (2.9)	66 (38.2)	102 (59.0)
Physical sequelae	17 (9.8)	99 (57.2)	57 (32.9)
Challenges and problems related to sexuality and intimacy?	52 (30.1)	88 (50.9)	33 (19.1)
Mental sequelae?	3 (1.7)	49 (28.3)	121 (69.9)
Social consequences?	27 (15.6)	79 (45.7)	67 (38.7)
Existential considerations?	19 (11.0)	66 (38.2)	88 (50.9)
Challenges and lifestyle-related issues?	6 (3.5)	58 (33.5)	109 (63.0)
To what extent does the written information you receive from specialised hospital departments met your need for knowledge about:			
The patient’s cancer diagnosis?	11 (6.4)	49 (28.3)	113 (65.3)
The patient’s cancer treatment?	28 (16.2)	75 (43.4)	70 (40.5)
Possible side effects and long-term effects of the patient’s cancer diagnosis and treatment?	113 (65.3)	46 (26.6)	14 (8.1)
Who is responsible for cancer follow-up?	67 (38.7)	73 (42.2)	33 (19.1)

More than half of the GPs assessed they were qualified to assess mental sequelae (70%), existential considerations (51%), impact on lifestyle (63%), and comorbidities (59%). On the other hand, only 19% and 33% of the GPs, respectively, reported competences regarding sexual and physical sequelae (Table 2).

Some 65% of the GPs assessed that the written information from hospital departments met their need for knowledge about the patient’s cancer diagnoses, while only 8% had sufficient knowledge about possible side effects and long-term effects of their patient’s cancer treatment (Table 2).

Female GPs were less likely to report competences regarding physical sequlae (adjusted odds ratio [OR _adj_] 0.4, 95% confidence incidence [CI] = 0.2 to 0.7), sexual sequelae (OR_adj_ 0.5, 95% CI = 0.3 to 0.9), and mental sequelae (OR_adj_ 0.4, 95% CI = 0.2 to 0.8), and were more likely to report competences regarding comorbidities (OR_ajd_ 2.4, 95% CI = 1.2 to 4.6) than male GPs. GPs from partnership practices were more likely to report competences assessing mental sequelae (OR_adj_ 2.4, 95% CI = 1.2 to 5.0), while no statistically significant associations were found in relation to seniority in practice or region (Table 3). Crude analyses are shown in Supplementary Table S1.

**Table 3.** Associations between GP and practice characteristics and the GP’s self-assessment of competences regarding late effects of cancer. Higher OR indicates higher self-asesssment of competences

	Other somatic diseases and treatment	Physical sequelae	Sexual sequelae	Mental sequelae	Social sequelae	Existential considerations	Lifestyle challenges
	Adj OR (95% CI)	Adj OR (95% CI)	Adj OR (95% CI)	Adj OR (95% CI)	Adj OR (95% CI)	Adj OR (95% CI)	Adj OR (95% CI)
**Sex**							
Male	1	1	1	1	1	1	1
Female	**2.4 (1.2 to 4.6**)*	**0.4 (0.2 to 0.7**)*	**0.5 (0.3 to 0.9**)*	**0.4 (0.2 to 0.8**)*	0.7 (0.4 to 1.4)	0.9 (0.5 to 1.7)	0.6 (0.3 to 1.1)
**Seniority in practice**							
<10 years	1	1	1	1	1	1	1
10–19 years	0.7 (0.3 to 1.5)	1.6 (0.7 to 0.5)	1.1 (0.5 to 2.2)	1.5 (0.7 to 3.5)	1.3 (0.6 to 2.6)	1.4 (0.7 to 3.0)	0.9 (0.4 to 1.9)
≥20 years	0.5 (0.3 to 1.2)	1.4 (0.6 to 3.3)	1.2 (0.6 to 2.6)	1.2 (0.5 to 2.9)	0.9 (0.4 to 1.9)	1.0 (0.5 to 2.3)	0.9 (0.4 to 2.1)
**Practice type**							
Single-handed practice	1	1	1	1	1	1	1
Partnership practice	0.5 (0.3 to 0.9)	0.9 (0.5 to 1.7)	1.1 (0.6 to 1.9)	**2.4 (1.2 to 5.0**)*	1.4 (0.8 to 2.5)	1.8 (1.0 to 3.3)	1.5 (0.4 to 2.9)
**Region**							
Capital Region of Denmark	1	1	1	1	1	1	1
Region of Southern Denmark	1.2 (0.7 to 2.2)	0.9 (0.5 to 1.7)	0.8 (0.5 to 1.5)	1.0 (0.5 to 2.1)	0.8 (0.5 to 1.5)	0.8 (0.5 to 1.5)	1.0 (0.5 to 1.8)

Bold and asterisk indicates significance at 5% level. Adjusted for: sex, seniority in practice, practice type, and region.

Adj OR = adjusted odds ratio. CI = confidence interval. OR = odds ratio.

In total, 28% of the GPs reported that they had implemented a systematic organisation of follow-up cancer care in their clinic. Examples of the systematic efforts from the free-text boxes appears in Table 4.

**Table 4.** Systematic efforts for follow-up cancer care in general practice, reported by the GPs (*n* = 173)

	Yes, *n* (%)	No, *n* (%)
Has your practice introduced systematic efforts for patients with cancer?	49 (28.3)	124 (71.7)
Quotes from the GPs describing the systematic efforts		
'*We write a letter/e-mail or call the patient, when we become aware of the diagnosis. Always offers time for follow-up in general practice. In the event of non-appearance, we give the patient a call.*’ '*Instructions phrased approximately: the one who reads the discharge summary/correspondence and becomes aware that a patient has been diagnosed with cancer or* [their cancer has been diagnosed as terminal]*, puts a reminder on the calendar of the usual GP. This GP then contacts the patient and clarifies whether there is a need for follow-up now or later and evaluate the resources around the patient.’* *‘A list of cancer patients, which is reviewed every six months.*’ *‘Letter to the patient that we know they have been diagnosed with cancer and that they can contact us in need of help. If we have not heard from them, we call them after one month.’* *‘Either a standard letter (adjusted Danish Society of General Practice template) or a call, depending on previous knowledge of the patient. However, it varies when in a cancer course, the contact is established.’* *‘We either call or write to patients with newly diagnosed cancer, and follow-up on the cancer patients we already know.’* *‘As soon as I see in a discharge summary that a patient has been diagnosed with cancer, I send a letter, with an invitation to a conversation together with relatives, either at home or in the clinic.’* *‘We call the patients, when we see a discharge summary with a newly diagnosed cancer, change to palliative care treatment, or information that the cancer has metastasised.’* *‘I contact them by email. In that way they can read the email when they have the time and surplus energy and write me back, when they can handle it. Unfortunately, I have just found out that you are not allowed to do that. At least one must not charge a fee for it. However, I still believe that regular follow-up is the right thing to do. Then I must just waive the fee.’* '*A palliation list, which is reviewed by the nurse, followed by phone calls and/or written offers of a consultation or visit.*’ *‘In relation to diagnosis, we always send a letter inviting the patient to take contact when needed and inform that we follow their treatment at the hospital “from behind the scenes”.’*		

Most GPs described efforts to get in contact with the patients and invite them to a consultation. No associations between GP and practice characteristics and having implemented a systematic effort were found in the adjusted analyses (Table 5).

**Table 5.** Associations between GP and practice characteristics and having implemented systematic efforts for follow-up cancer care (*n* = 173)

	Systematic efforts	
	Crude OR	Adj^a^ OR (95 % CI)
**Sex**		
Male	1	1
Female	0.5 (0.3 to 1.1)	0.7 (0.3 to 1.4)
**Seniority in practice**		
<10 years	1	1
10–19 years	1.1 (0.5 to 2.4)	1.0 (0.4 to 2.2)
≥20 years	3.0 (1.2 to 7.3)	2.3 (0.9 to 6.2)
**Practice type**		
Single-handed practice	1	1
Partnership practice	0.6 (0.3 to 1.3)	0.8 (0.4 to 1.7)
**Region**		
Capital Region of Denmark	1	1
Region of Southern Denmark	0.6 (0.3 to 1.2)	0.6 (0.3 to 1.2)

Bold indicates significance at 5% level. ^a^Adjusted for: sex, seniority in practice, practice type, and region.

Adj OR = adjusted odd ratio. OR = odd ratio.

## Discussion

### Summary

We explored the organisation of follow-up cancer care in Danish general practice and analysed GPs’ self-assessment of competences in addressing late effects. Less than one in three general practices have implemented systematic follow-up cancer care in their clinic. Most GPs assess their own competence high with respect to assessing late effects in terms of mental sequelae, existential considerations, and impact on comorbidities and lifestyle. However, the GPs assess their competences lower with respect to physical and sexual sequelae. Female GPs more often report high competences regarding comorbidities than male GPs, while they rate their competences lower regarding physical, sexual, and mental sequelae. Competences related to assessment of mental sequelae are higher among partnership practices compared with single-handed practices.

### Strengths and limitations

The random sample representative of Danish general practices is a strength of the study, yet the participation rate of 35% is lower than desired, but in line with previous survey studies among Danish general practices.^9^ Although the responders were fairly comparable with Danish general practices regarding sex, age, and practice type,^9,10^ we cannot rule out non-responder bias. The responding GPs may be more engaged and interested in the topic of follow-up cancer care and palliative care, which could lead to an overestimation of the self-assessment of competences and systematic follow-up. If this is the case, the possible overestimation in fact emphasises the need for more focus on the knowledge and management of late effects in general practice. Further, the predefined closed answer categories may be a limitation of the study. Therefore, we offered the possibility of elaborating on the organisation of follow-up strategies in free-text boxes. A thorough qualitative thematising and analyses of the text boxes are beyond the scope of this study, but we believe the presented statements provide valuable insights and nuances.

We chose an ordered logistic regression model, as it allows us to include all categories for the outcome variable in an ordered manner, under the assumption that the ‘distance’ between the categories is equal across the range, that is, fulfilling the proportional odds assumption.

### Comparison with existing literature

Previous studies have targeted the patient need of attention to follow-up cancer care, and potential lack of professional awareness from their GPs.^11–15^ Patients with cancer suffer from both specific late effects that are related to individual cancer sites and treatments, and more general late effects, including depression, fear of recurrence, cognitive impairment, sleep disturbances, fatigue, and pain issues.^3^ Compared with the specific late effects, these general symptoms and diseases are prevalent across all patients who have survived cancer and influence many patients in their daily life. Furthermore, the general late effects are often related, and patients rarely experience only one.^3^ According to an Australian review, a substantial number of patients who have survived cancer experience unmet needs, particularly in relation to psychosocial challenges,^16^ which are not adequately dealt with in the existing healthcare system.^4^ Moreover, many patients who have survived cancer are more likely to die from chronic, often preventable non-communicable disease, rather than from their malignancy.^17^ Therefore, lifestyle interventions targeted to each patient’s risk profile and personal preferences should also be prioritised, which is a task GPs are already familiar with.

A prerequisite for the GPs to play a sufficient role in the follow-up care is insight into the patient’s course of disease. Many patients have sparse contact with their GPs during their treatment at the hospitals, and the contact needs to be re-established after the end of treatment.^18,19^ Participating GPs in the present study reported to receive most information regarding patients in need of follow-up care from the hospital discharge cards or from the patients. However, fewer than two out of three GPs reported that the written information from the hospital met their need for knowledge about the patient’s cancer diagnoses, and <10% had sufficient knowledge about possible side effects and late effects of their patient’s cancer treatment. Being a patient can be a daunting and overwhelming experience, particularly for those who are less familiar with the organisation of the healthcare system or who may have had negative experiences in the past. Far from all patients seek help themselves,^19^ and some of these patients may benefit from systematic follow-up from their GP. The literature points to the fact that health literacy challenges may lead to misinterpretation of symptoms and postponing or omitting relevant healthcare-seeking,^20,21^ and individuals with health literacy challenges may therefore benefit more from continuity and easy access to consultations with the same doctor or nurse every time they contact general practice.

### Implications for research and practice

The increasing prevalence of patients who have survived cancer has reinforced the need for strategies for follow-up cancer care in general practice. We investigated the organisation of follow-up cancer care in Danish general practice and analysed GPs’ self-assessment of competences regarding patients who have survived cancer and late effects. Fewer than one in three Danish general practices have organised systematic follow-up cancer care, and GPs assess their competences as low with respect to physical sequelae and sexual challenges. This emphasises the need for more systematic organisation and focus on management of late effects in general practice, to ensure patients with or who have survived cancer have a more coherent and comprehensive follow-up in primary care.

Health systems are continually looking for ways of optimising resource usage, while maintaining quality of treatment and care.^22^ Future studies should address the transition from hospital treatment to follow-up in primary care, the importance of collaboration between healthcare providers, knowledge dissemination between the healthcare sectors, and involvement of patients to ensure that those who have survived cancer have adequate help regarding their late effects.

## Data availability statement

The datasets generated and analysed in the current study are available from the corresponding author on reasonable request, but restrictions apply to the availability of these data due to the data protection regulations from the Danish Data Protection Agency, and so are not publicly available. Access to data is strictly limited to the researchers who have obtained permission for data processing. This permission was given to the Research Unit of General Practice, Department of Public Health, University of Southern Denmark.
